# Exploring the Impact of Optimized Probiotic Supplementation Techniques on Diabetic Nephropathy: Mechanisms and Therapeutic Potential

**DOI:** 10.7759/cureus.55149

**Published:** 2024-02-28

**Authors:** Anindita Ghosh, Arti Muley, Archana S Ainapure, Aditi R Deshmane, Anu Mahajan

**Affiliations:** 1 Nutrition and Dietetics, Symbiosis Institute of Health Sciences, Symbiosis International (Deemed University), Pune, IND; 2 Beauty Wellness and Nutrition, Symbiosis Skills and Professional University, Pune, IND; 3 Clinical Nutrition, Indian Institute of Food Science and Technology, Aurangabad, IND

**Keywords:** probiotic, lipid profile, diabetes mellitus, diabetic nephropathy, antioxidant

## Abstract

Worldwide, diabetic nephropathy (DN) is a significant contributor to end-stage renal failure and chronic kidney disease. Probiotic supplementation has recently gained popularity as a potential nutritional therapy in several clinical trials aimed at improving renal function, inflammation, oxidative stress, dyslipidemia, glycemic control, and inflammation. However, they still need to undergo a thorough assessment of DN. It is crucial that the optimal dosage, duration, and combination of probiotic strains administered for the purpose of slowing down the advancement of DN be assessed. Based on the available publications, including relevant randomized controlled trials, systematic reviews, and meta-analysis from 2013-2023 from search engines like MEDLINE (PubMed), Scopus, and Web of Science, a literature review was generated using the keywords "gut microbiota," "gut microbiome," "diabetic kidney disease," "diabetic nephropathy," "probiotic," and "prebiotic." Multiple clinical trials focusing on probiotic administration techniques revealed changes in renal, glucose, and lipid biomarkers. Probiotic supplementation using *Bifidobacterium bifidum*, *Lactobacillus acidophilus,* and *Streptococcus thermophilus* for 12 weeks indicated a reduction in glycosylated hemoglobin, fasting blood glucose, and the microalbuminuria/creatinine ratio. Multispecies as well as single-species probiotic administration containing *Lactobacillus, Bifidobacterium, *and* Streptococcus thermophilus spp.* greater than 4*10^9^ colony forming units (CFU)/day for 8-12 weeks in DN patients improves renal metabolic markers and reduces the progression of disease patterns. Optimal supplementation techniques of probiotics in conjunction with prebiotics and synbiotics in DN benefit glycaemic control, renal function, blood lipid profile, inflammation, and oxidative stress. Future randomized controlled trials supplementing specific probiotics coupled with prebiotics and synbiotics, with larger sample sizes and longer follow-up times, will generate more reliable findings for the impact of probiotic supplementation on DN.

## Introduction and background

Diabetic kidney disease (DKD) is a precarious chronic vascular condition, complicating the pathways of kidney disease and paralleling it to end-stage kidney disease. As of 2021, around 537 million adults (20-79 years old) globally have diabetes; by 2045, that figure is expected to rise to 783 million [1]. An estimated 40% of diabetic patients will eventually experience kidney problems [2]. About 30% to 40% of the world population with diabetes end up with diabetic nephropathy (DN) due to persistent poor glycemic and blood pressure control [3]. While the exact cause of the poor glycemic management that results in diabetic neuropathy is unknown, metabolic and genetic reactions akin to insulin resistance, hyperglycemic states, and autoimmunity are thought to be involved ​[4]. The structural and functional changes of the kidney due to the consistent hyperglycemia increase the incidence of proteinuria, hypertension, and a reduction in the capacity of the nephrons to participate in the filtration process, further progressing the kidney disease. Recent research has suggested that gut microbiome alterations may influence DN development [5]. In people with gut microbiota dysbiosis, higher azotemia and the release of toxicants such as ammonia, amines, indoles, thiols, and phenols have been linked to an increase in dangerous bacteria in the intestine [6]. As researchers continue to explore novel strategies to manage DN, probiotic supplementation is an emerging topic of interest, and the optimization of the administrative dosage and duration of the specific and significant probiotic strains are essential elements to reach the goal of non-progression of DN to end-stage renal disease.

Dysbiosis in DN

An array of microorganisms, such as bacteria, viruses, fungi, and other microbes, indicates a healthy gut microbiome. Microbial diversity is frequently reduced due to dysbiosis, with some species predominating in the gut ecosystem while others decline. The ability of the microbiome to carry out vital tasks like metabolizing food components, regulating immune responses, and guarding against infections may be jeopardized by this imbalance. The gut epithelial barrier is an essential interface between the microbiome and the host's internal environment. The integrity of the gut barrier is compromised by dysbiosis, resulting in a leaky gut and increased intestinal permeability, allowing toxins and microbial products to enter the bloodstream. The gut epithelial barrier is an essential interface between the microbiome and the host's internal environment. This phenomenon may result in immunological activation and systemic inflammation and contribute to the pathogenesis of various diseases.

Gut microbiota alterations have been increasingly recognized as essential contributors to the pathogenesis and progression of various diseases, including DKD. Dysbiosis, reduced microbial diversity, altered bacterial taxa, gut barrier dysfunction, and changes in gut-derived metabolites leading to the trigger of the renin-angiotensin system collectively contribute to DKD [7]. Novel therapeutic approaches targeting the gut microbiota to prevent or manage DKD require comprehensive knowledge about the intricacies of the pathways involved between them. Earlier studies recorded the reduced gut microbial profiles between patients with DKD and healthy controls, with specific microbial taxa showing associations with DKD severity [8,9]. DKD patients exhibit changes in the relative abundance of several bacterial groups. In DKD patients, a comparative prevalence of the genus *Escherichia/Shigella, Proteobacteria *spp*.*, and the scarcer presence of *Firmicutes*,* Bifidobacterium*,* and Bacteroidetes *spp*.* was prevalent when compared to non-diseased individuals [10]. They are prone to *Faecalibacterium prausnitzii* and *Roseburia* sp., which have a lower capacity for butyrate production [8]. These changes in gut microbial composition can have profound implications for DKD progression.

DKD is associated with increased gut barrier dysfunction, which may contribute to the displacement of microbial products into the total blood flow. Elevations of circulating lipopolysaccharide present in the outer membrane of pathogenic bacteria are found in DKD patients, suggesting increased gut barrier dysfunction [11,12]. This translocation can trigger an immune response and promote inflammation, further exacerbating kidney damage, increasing gut permeability, and alterations in tight junction protein expression, indicating compromised gut barrier function [11].

**Figure 1:**
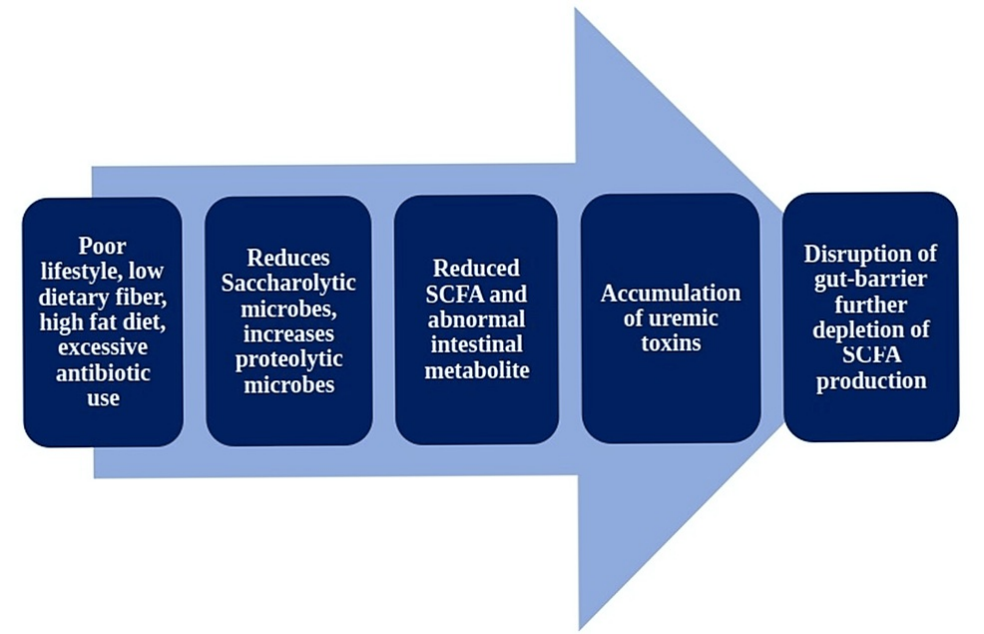
Schematic diagram proposing the mechanism of gut dysbiosis in DKD patients SCFA: short-chain fatty acid, DKD: diabetic kidney disease Image Credit: Author

Fermented dietary fibers and complex carbohydrates stabilize the gut microbiota by producing short-chain fatty acids (SCFAs) like butyrate, acetate, and propionate. Dysbiosis in DKD can lead to decreased SCFA production and impaired gut barrier function, allowing microbial products and inflammatory molecules to be translocated in the blood. This phenomenon, called metabolic endotoxemia, contributes to the low-grade chronic inflammation observed in DKD and may exacerbate kidney damage [13]. Fecal SCFA profiles in DKD patients found reduced levels of particularly butyrate. Butyrate is prime for limiting inflammation and protecting against kidney damage [14]. Figure 1 explains the mechanism of gut dysbiosis in DKD patients.

Role of probiotics in DN

To prevent the progression of DN, the first and foremost target becomes confronting dysbiosis. Though reduction of uremic toxins can be achieved by following a therapeutic diet and lifestyle, the sustainability of these long-term diets is questionable. Thus, probiotic supplementation has become one of the most reliable therapeutic techniques [15]. The present paper focuses on their positive effects on gut health, immune function, and metabolic disorders. In the contemporary period, accumulating research evidence suggests a potential correlation between probiotic supplementation and improved outcomes in DN [4]. This correlation becomes much more robust when probiotics are used in synergies with prebiotic and fecal transplants [16]. Numerous studies have shed light on the effect of probiotic supplementation on DN and the lowering of renal function markers [17]. However, only some researchers claim the specific composition, dosage, and duration of the particular or mixed probiotic strains used as the remedy [18]. In traditional medicines, a combination of specific herbs like *Astragalus membranaceus* and *Salvia miltiorrhiza* has been proven to improve the gut microbial count, reducing the progression of DKD [19]. Different probiotic strains have their process of operation by producing anti-bacterial elements, interfering with cellular bindings, promoting tight junction integrity, impairing toxin receptor activity, and calibrating PH and nutrient rivalry [20]. Therefore, direct administration of probiotics in accurate dosages, duration, and form makes it a significant step in controlling DKD and its progression to end-stage kidney disease [21].

**Figure 2:**
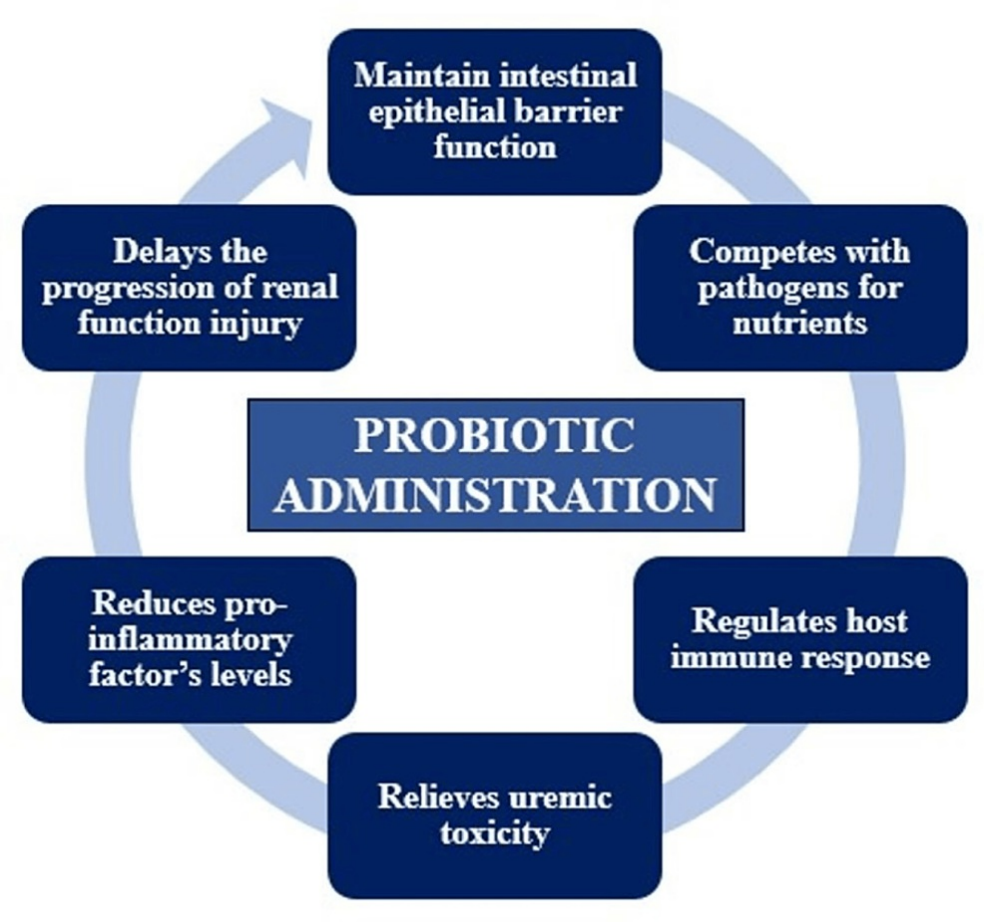
Proposed probiotic action on delaying progression of renal injury Image Credit: Author

Probiotics probably have a multifactorial mode of action on DN. Probiotics have been shown to modulate microbiota content, reduce chronic low-grade inflammation, and enhance gut mucosal barricade function-factors that are closely associated with the pathogenesis of DN [22]. Figure 2 explains the mechanism of action of probiotics on DN.

While the existing evidence suggests a potential correlation between probiotic supplementation and improved outcomes in DN, further research is needed to fully understand the optimal strains, dosages, and duration of probiotic intervention.

Objectives

In this article, we aim to examine the existing literature on the relationship between probiotic supplementation and DN and answer the research question on probiotic intake patterns, probiotic dose, and duration of intervention and its effect on the biomarkers of DN. By reviewing relevant studies and considering the potential mechanisms of action, we aim to contribute to the ongoing discussion surrounding probiotic usage as a possible adjunctive strategy for managing DN.

## Review

Methodology

**Figure 3:**
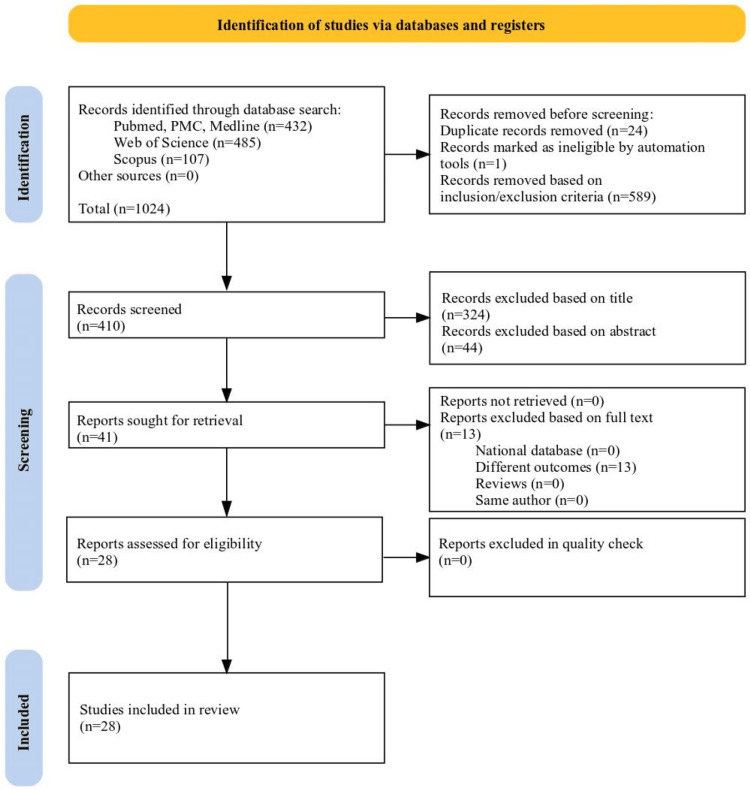
PRISMA flow diagram for methodology PRISMA: Preferred Reporting Items for Systematic Reviews and Meta-Analyses

An extensive literature search to find eligible studies in MEDLINE (PubMed), Web of Science, and Scopus was performed using combinations between particular medical subject headings (MeSH) terms: "gut microbiota," "gut microbiome," "diabetic kidney disease," "diabetic nephropathy," "probiotic," and "prebiotic" from 2013 to 2023. Prebiotic was used as a MeSH term as it promotes the reproduction and metabolism of intestinal probiotics, which reduces the progression of DN. Prebiotic administration improved the sustainability of the probiotic strains in the intestine for a longer duration. No language filters or other specifications were added to the search strategy. The comprehensive search plan for all databases and sources found is described in Figure 3.

A multistep approach was used to assess the retrieved references for eligibility, as depicted in Figure 3, using a PRISMA flow diagram [23]. In the first step, the titles and abstracts of articles for inclusion and exclusion criteria were evaluated. In the next step, the full text of the studies that met the eligibility criteria based on title and abstract was appraised. Several inclusion criteria were pre-defined and applied for eligibility assessment: (1) randomized clinical trial (RCT) design and non-RCT design; systematic reviews and meta-analysis; (2) DN patients without restriction on age or medical conditions intervened using probiotics; and (3) assessment of renal function, glucose control, lipid profile, and inflammation score was reviewed on probiotic, prebiotic, and symbiotic supplementation. In addition, some critical exclusion criteria were established, including (1) animal studies; (2) articles in languages other than English were excluded; (3) literature without full text or literature exhibiting insignificant outcomes; (4) unpublished data; (5) studies available only in abstracts, case reports, editorials, missing data, and the inability to extract data regarding the population enrolled and outcomes investigated. Additionally, we disregarded studies that used non-bacterial probiotics or other biomarkers and focused on other illnesses or gestational diabetes. After checking the eligibility based on the inclusions and exclusions, 28 publications were selected for further study and analysis, adhering to the research objective.

Results

Prebiotics and probiotics effectively reduce uremia and blood glucose, restructure the gut microbiota, and mitigate inflammation and oxidative damage in chronic kidney disease (CKD) [6,24]. Oral administration of probiotics has been found to have anti-phlogistic implications in acute kidney injury through the advancement of SCFAs and alleviating chronic renal interstitial fibrosis [9]. Several RCTs have been conducted to investigate the outcome of probiotics in type 2 diabetes mellitus (T2DM)-induced DKD. Of the 28 trials, 14 showed a significant change in the kidney, glucose, and lipid biomarkers. A common endpoint in clinical trials assessing DKD interventions is the estimated glomerular filtration rate (eGFR). As eGFR varies over time, it can be used as an indirect measure to evaluate the effectiveness of treatments meant to maintain or enhance kidney function in DKD patients. One of the main objectives of DKD treatment is frequently to stabilize kidney function or slow the decline in eGFR. Compared to conventional soy milk, multi-strain probiotic consumption reduced serum interleukin-18 (IL-18), improved kidney function, and enhanced antioxidant factors and enzymes [25-27]. Moreover, the latest meta-analysis indicated soy milk supplements, capsules, and sachets of probiotics significantly improved glucolipid metabolism, ameliorated renal impairment, and reduced oxidative damage in DKD patients [28].

Kidney Biomarkers

Serum creatinine (sCr) levels: The impact of pro/pre and synbiotic supplementation therapy on sCr was observed among two significant clinical trials, with a distinct difference in both groups. Probiotic soy milk supplementation for eight weeks with a daily dose of 4*10^9^ colony forming units (CFU) *Lactobacillus plantarum A7* significantly decreased sCr levels in a group of 40 T2DM with DN (T2DM-DN) patients in comparison to usual standards in a control group receiving conventional soy milk [25]. In another study, multispecies probiotic administration of dosage 8 × 10^9^ CFU/day for 12 weeks combining *Lactobacillus acidophilus ZT-L1*, *Bifidobacterium bifidum ZT-B1*, *Lactobacillus plantarum ZT-B1*, *Lactobacillus reuteri ZT-Lre*, and *Lactobacillus fermentum* *ZT-L3* lowered sCr levels very prominently in a different cohort of 60 DN patients (56 T2DM and four T1DM) where the control group was administered only starch [29]. Numerous other trials that were executed could not show much significance in proving a correlation between probiotic supplementation and a reduction of sCr levels [30].

Blood urea nitrogen (BUN): In a Malaysian study, 12 weeks of multiple-strain probiotic preparation dispensation containing 6*10^10^ CFU/day of *Lactobacillus acidophilus*,* Lactobacillus casei*,* Lactobacillus lactis*,* Bifidobacterium bifidum*,* Bifidobacterium longum*,and* Bifidobacterium infantis* mixed with water were responsible for a significant decrease (4.26 ± 1.29 at baseline to 4.04 ± 1.04 mmol/L at end-of-trial) in BUN levels; when compared, a rise in BUN was observed in the control groups [31]. Another multi-strain formulation using 8 × 10^9^ CFU/day of *Lactobacillus acidophilus ZT-L1*,* Bifidobacterium bifidum ZT-B1*,* Lactobacillus reuteri ZT-Lre*,and* Lactobacillus fermentum ZT-L3* considerably lowered the BUN levels to −3.5 ± 5.8 mg/dL from baseline in the experimental unit upon 12-week administration in contrast to the group under control [29]. A dosage of probiotic strains >4*10^9^ CFU/day was used in five studies, including 406 participants (203 intervention; 203 controls) showing a significant effect on lowering BUN [30].

Urinary albumin/creatinine ratio (Alb/Cr): Two RCTs displayed a notable reduction in urinary Alb/Cr ratio among 116 participants (62 intervention; 54 control). Administration of a single species probiotic by Abbasi et al. caused a change of 16.5 ± 12.2 mg/g from baseline urinary Alb/Cr, as opposed to 5.7 ± 15.04 mg/g in controls who received conventional soy milk [25]. Therapeutic use of a multispecies probiotic strain composed of 3.2 × 10^9^ CFU/day of *Bifidobacterium bifidum*,* Lactobacillus acidophilus*,and* Streptococcus thermophilus* by Jiang et al. in a Chinese study consisting of 76 T2DM-DN patients showed a reduction in urinary Alb/Cr ratio from 101.60 ± 22.17 to 67.53 ± 20.11 mg/g from the baseline after the conclusion of the study [32].

eGFR: A notable rise in eGFR from a baseline of 15.9 ± 10.8 mL/min (1.73 m2/min) was found in the experimental group on probiotic soy milk supplementation for 12 weeks; a minor positive impact was also reflected in the control group [25]. Administration of multispecies probiotic strain formulations also showed a significant rise in the eGFR value by 8.3 ± 17.3 mL/min from baseline compared to the group under control [29]. Only these two clinical trials could show significant results out of seven RCTs. The other five RCTs, including 190 experimental participants and 182 controls, failed to show any statistical inter-correlation of GFR with probiotic supplementation even after performing any sub-group analysis for heterogeneity [33].

Reduction in uremic toxins: Indoxyl-sulfate, p-cresyl sulfate, indole-3-acetic acid, trimethylamine N-oxide, and phenylacetylglutamine are gut-derived uremic toxins linked to cardiovascular disease, CKD mortality, and other end-organ toxicity [28]. *Escherichia coli* metabolizes dietary tryptophan by the enzyme tryptophanase, producing uremic toxins like indoxyl-sulfate and indole-3-acetic acid. Indoxyl-sulfate binds with albumin, is normally excreted in urine, and, therefore, cannot be effectively cleaned by conventional hemodialysis [33]. In two RCTs, a significant improvement in the cystatin C levels on probiotic administration to 130 DKD patients (65 intervention; 65 control) was found with no heterogeneity. Probiotic supplementation could reduce p-cresyl sulfate, a type of urea toxin caused by dysbacteriosis, but no significant role was found in the reduction of BUN and sCr [32].

Electrolyte balance: Three RCTs investigated the probiotic’s outcome on potassium levels among 236 DKD patients (intervention, 118; control, 118). The pooled results revealed no significant decrease in potassium levels in contrast to the groups under control. No statistically meaningful subgroup analysis was conducted based on the intervention timeline, probiotic dose, or consumption patterns. With 196 participants, two RCTs investigated the probiotic’s outcome on sodium levels (intervention, 98; control, 98). Data analysis revealed that probiotic administration caused a notable decline in sodium levels in patients with DN [28].

Glycemic Biomarkers

In seven RCTs, five studies showed considerable impact on the administration of probiotics in lowering plasma glucose levels during fasting, the homeostatic model assessment for insulin resistance (HOMA-IR) among 346 DKD cases (intervention, 177; control, 169), but no statistically significant effect on glycated hemoglobin (HbA1C), quantitative insulin-sensitivity check index was present. The result was more notable when mixed-culture probiotics were used at a dosage of >4*10^9^ CFU/day [34]. A significant reduction in insulin with no effect on HOMA-IR was found in the study of AbdelQadir et al. [35]. The other three randomized controlled trials with 180 participants (intervention, 90; control, 90) could also not show any positive implications. Subgroup investigation revealed that the effects of HOMA-IR were more evident among instances with multiple species of probiotics and probiotic doses greater than 4*10^9^ CFU/day [28]. In two RCTs among 101 participants (intervention, 87; control, 79), no significant change was found in glycemic control (post-involvement value 17.13 ± 6.05 mmol/L vs. pre-involvement value 19.00 ± 6.41 mmol/L), two-hour postprandial blood glucose, and HbA1c (%) (7.92 ± 1.21% post vs. 8.25 ± 2.03% pre-participation) after probiotic administration of 3.2 *10^9^ CFU/day through a 12-week timeline). In contrast, depletion of fasting plasma glucose (7.81 ± 2.77 mmol/L following therapy vs. 10.68 ± 3.24 mmol/L pre-therapy) was noted [32]. In a randomized, double-masked cross-over clinical trial comprising 62 people with diabetes, a 9-gram synbiotic food containing *Lactobacillus sporogenes* (1 *10^7^ CFU) as a heat-resistant probiotic strain, 0.04 g inulin with 0.38 g isomalt as a prebiotic, 0.36 g sorbitol, and 0.05 g stevia as sweetener per 1 g was given for six weeks. Reduction in high-sensitivity C-reactive protein (hs-CRP) levels, glutathione (GSH) levels, and a substantial drop in insulin levels in the serum (variations to the initial value: -1.75 ± 0.60 vs. +0.95 ± 1.09 μIU/mL) were the results of supplementation [36]. Twelve weeks of intervention with 25 g of probiotic honey containing 10^8^ CFU/g of *Bacillus coagulans T11 (IBRC-M10791)* in 30 DN patients in a double-blind RCT revealed extensively lower serum insulin levels (−1.2 ± 1.8 vs. −0.1 ± 1.3 μIU/mL, p=0.004), HOMA-IR levels (−0.5 ± 0.6 vs. 0.003 ± 0.4, p=0.002), and drastically increased quantitative insulin sensitivity check index (+0.005 ± 0.009 vs. −0.0007 ± 0.005, p=0.004) compared to the other 30 DN patients who received 25 g of non-probiotic honey per day [30].

Lipid Biomarkers

In a recent clinical trial in Thailand, 50 participants with hypercholesteremia were randomly trialed for three months to consume *Lactobacillus paracasei TISTR 2593.* In the placebo group, maltodextrin capsules were given for consumption for the same period as the probiotic strain. After the trial period ended, it was found that the probiotic administration had lowered the low-density lipoprotein cholesterol (LDL-c) and reduced oxidative stress and inflammation in Thai individuals, reducing the risk of atherosclerosis, which stands as a significant complication of DN [17]. The systemic literature review and cumulative findings by Dai et al. revealed a decline in DKD-worsening risk variables like triglyceride (TG), total cholesterol (TC), and LDL-c when a single-strain probiotic was consumed at 4*10^9^ CFU/day for eight weeks [28]. The longer the intervention, the more significant the reduction of LDL-c. This study focused on the role of probiotics in the breakdown of indigestible carbohydrates derived from food and increased SCFA production, which is a preliminary factor for controlling blood lipid profiles. Probiotics also play a role in the slower and reduced absorption of dietary cholesterol and bile acid by the small intestine. AbdelQadir et al. discovered no improvements in TG, TC, LDL-c, or high-density lipoprotein cholesterol (HDL-c) on multispecies probiotic administration of the exact dosage and duration [35]. In seven RCTs with 340 participants, lipid biomarkers such as TG, TC, LDL-c, and HDL-c were reduced marginally on probiotic administration, but no statistical significance was established [37]. Single-species probiotic formulations were much more capable of lowering TC and LDL-c. However, there was no precise mechanism for the effect of different doses and intervention durations on the lipid profile, requiring further investigation. In 2017, 10 RCTs comprising 297 patients in the treatment group and 294 patients in the control group showed a reduction of total cholesterol, triglycerides, low-density lipoprotein, and systolic blood pressure on probiotic administration. In contrast, high-density lipoprotein increased [38]. In a randomized, double-masked, placebo-controlled trial with 20 senior citizens (10 cases; 10 controls), a symbiotic beverage containing 10^8^ CFU/mL of *Lactobacillus acidophilus*, 10^8^ CFU/mL of *Bifidobacterium bifidum,* and 2 gm oligo fructose was administered to the experimental group daily for 30 days, which resulted in an increased HDL-c count [39].

Inflammatory Biomarkers

In an interventional study, 48 patients with DN were randomly made to ingest 200 ml/day of soy milk containing probiotics, while the control group received only soy milk. Following an eight-week intervention, the standard methodology was followed to test malondialdehyde (MDA), 8-iso-prostaglandin F2, oxidized GSH, total antioxidant capacity, GSH, GSH peroxidase, and GSH reductase as indicators of oxidative stress. The intervention group showed a considerable rise in the GSH, GSH peroxidase, and reductase activity levels and a lower oxidized GSH quantity than the control group [27]. A meta-analytic study comprising 10 RCT probiotic dosages of more than 5 billion CFU/day for 12 weeks increased the total antioxidant capacity and GSH levels, simultaneously reducing hs-CRP and MDA levels [9]. In a related study, Mazruei Arani et al. found that administering to 30 DN patients 25 grams of probiotic honey for 12 weeks containing 10^8^ CFU/g of *Bacillus coagulans T11 *(*IBRC-M10791*) improved the levels of serum hs-CRP and plasma MDA. In comparison to the control group of 30 DN patients who received plain honey without a probiotic, the probiotic group showed a lowering of hs-CRP levels (−1.9 ± 2.4 vs. −0.2 ± 2.7 mg/L, p=0.01) and MDA levels (−0.1 ± 0.6 vs. +0.6 ± 1.0 μmol/L, p=0.002) [30].

Table 1 clearly explains the type, dosage, frequency, trial length, and effects of probiotics on inflammatory, lipid, glucose, and kidney biomarkers that affect the course of DKD.

**Table 1:** Effect of probiotic type, dosage, and duration on kidney, glucose, and lipid biomarkers RCT: randomized clinical trial; T2DM: type 2 diabetes mellitus; T1DM: type 1 diabetes mellitus; CFU: colony forming units; BUN: blood urea nitrogen; hs-CRP: high-sensitivity C-reactive protein; WBC: white blood cell; eGFR: estimated glomerular filtration rate; HbA1c: glycated hemoglobin; TG: triglyceride; TC: total cholesterol; T2DM-DN: type 2 diabetes mellitus with diabetic nephropathy; sCr: serum creatinine; IL: interleukin; SSA: serum sialic acid; NGAL: neutrophil gelatinase-associated lipocalin; Cyst-C: cystatin C; sTNFR1: soluble tumor necrosis factor receptor 1; FBG: fasting blood glucose; HOMA-IR: homeostatic model assessment for insulin resistance; MDA: malondialdehyde; GSH: glutathione; HDL-c: high density lipoprotein; urinary Alb/Cr: urinary albumin creatinine ratio; TAC: total antioxidant capacity; LDL-c: low-density lipoprotein; Na: sodium; TNF: tumor necrosis factor; GLP-1: glucagon-like peptide-1

Author details	Study design	Patient profile	Types of probiotic	Intervention	Dosage and frequency	Trial duration	Changes in the biomarkers	Outcome
Wang et al., 2021 [8]	A systematic review and meta-analysis	7 RCTs (456 patients)	Multi-strain microbial cell preparation	Probiotic doses greater than 5 billion CFU/day	Soy milk, probiotic capsule, and tablet	8 weeks	↓BUN, sCr, MDA, hs-CRP, ↑eGFR	Delayed CKD progression
Miraghajani et al., 2018 [26,27]	Parallel RCT	48 T2DM-DN patients (case 24; control 24)	Single strain	Probiotic soy milk containing *Lactobacillus plantarum* A7 (2 × 10^7^ CFU/mL)	200 mL/day	8 weeks	↓Serum NGAL, Cys-c, ↑sTNFR1 (ng/mL)	Improved renal function
Dai et al., 2022 [28]	Systematic review and meta-analysis	10 RCTs, 552 participants	Single and multi-strain microbial cell preparation	<4*10^9^ CFU/day mixed culture probiotics	Microbial cell preparation, probiotic soy milk, probiotic capsule, and tablet	6, 8, and 12 weeks	↓sCr, Cys-c, urinary Alb/Cr, serum Na, BUN	Reduced inflammation and oxidative stress, improved kidney, lipid, and glucose biomarkers
Abbasi et al., 2017 [25]	Double-blind RCT	44 T2DM-DN patients from Iran (case 22; control 22)	Single strain	Probiotic soy milk containing *Lactobacillus plantarum* A7 (2 × 10^7^ CFU/mL)	200 mL/day	8 weeks	↓sCr (mg/dl), ↑eGFR	Improved glomerular function
Mafi et al., 2018 [29]	Placebo-controlled RCT	60 T2DM-DN (case 30; control 30)	Multi-strain microbial cell preparation	Supplemental form in capsule *Lactobacillus acidophilus* strain ZT-L1, *Bifidobacterium bifidum* strain ZT-B1, *Lactobacillus reuteri* strain ZT-Lre, and *Lactobacillus fermentum* strain ZT-L3 (each 2 × 10^9^) 8 x 10^9^ CFU/day	One capsule/day	12 weeks	↓FBG, HOMA-IR, hs-CRP, MDA, ↑insulin sensitivity, HDL-c, GSH	Improved glucose control, reduced cardiac risk, inflammation
Mazruei Arani et al., 2019 [30]	Randomized, double-blind, controlled clinical trial	60 patients with DN (case 30; control 30)	Viable and heat-resistant probiotic	Probiotic honey *Bacillus coagulans* T11 (IBRC-M10791) (10^8^ CFU/g)	25 g/day	12 weeks	↓serum insulin, HOMA-IR, hs-CRP, MDA, ↑total-/HDL-cholesterol	No significant improvement in the metabolic profiles
Firouzi et al., 2015 [31]	Double-blind, parallel RCT	136 T2DM (case 68; control 68)	Freeze-dried multi-strain microbial cell preparation	*Lactobacillus acidophilus*, *Lactobacillus casei*, *Lactobacillus lactis*, *Bifidobacterium bifidum*, *Bifidobacterium longum*, and *Bifidobacterium infantis* (6.0 × 10^10^ CFU/day total) mixed in water	Two sachets/day	12 weeks	↓BUN	Improved renal function
Jiang et al., 2021 [32]	Double-blind, placebo-controlled RCT	76 T2DM (case 34; control 42)	Multi-strain microbial cell preparation	*Bifidobacterium bifidum*, 1.2 x 10^9^ CFU, *Lactobacillus acidophilus* 4.2 x 10^9^ CFU, *Streptococcus thermophilus* 4.3 x 10^9^ CFU/day	One capsule/day	12 weeks	↓urinary Alb/Cr (mg/g), ↑HbA1C, FBG	Improved renal function and glucose control
Tarrahi et al., 2022 [34]	A systematic review and meta-analysis of clinical trials	7 trials with 346 DKD patients (intervention 177; control 169)	Single and multi-strain microbial cell preparation	Probiotic doses greater than 4*10^9^ CFU/day	Microbial cell preparation, probiotic	12 weeks	↓FPG and HOMA-IR, TNF, IL-6, and intestinal GLP-1	Reduction in insulin, Inflammatory index
Moravejolahkami et al., 2021 [37]	A systematic review and meta-analysis of clinical trials	7 RCT 258 T1DM individuals	A mixture of bacteria, single species	Probiotic doses greater than 4*10^9^ CFU/day	Microbial cell preparation, probiotic	12 weeks	↓TG, TC, LDL-c, ↑HDL-c	Outcome statistically non-significant
Bohlouli et al., 2021 [40]	A systematic review and meta-analysis of clinical trials	7 RCTs (220 patients)	Single and multi-strain microbial cell preparation	*Lactobacillus plantarum* A7, *Bacillus coagulans* T11 >5 billion CFU	Probiotic soy milk, capsule, sachet, honey	8 and 12 weeks	↓MDA, hs-CRP, ↑GSH, TAC	Improved renal function and glucose control
Akoglu et al., 2015 [41]	A hospital-based clinical study	142 hospitalized patients with acute gastroenteritis	Commercial strain microbial cell preparation	*Lactobacillus casei* Shirota (LcS) 10^8^/ml	130 ml (65 ml twice daily)	1 week	↓hs-CRP, WBC, ↑ eGFR	Reduction in leukocyte, inflammation, frequency of bowel movement, improvement in renal parameter
Ostadrahimi et al., 2015 [42]	Randomized double-blind placebo-controlled clinical trial	60 T2DM (case 30; control 30)	Multi-strain microbial cell preparation	Probiotic Kefir *Streptococcus thermophiles*, *Lactobacillus casei*, *Lactobacillus acidophilus,* and *Bifidobacterium lactis*	600 ml twice/day	8 weeks	↓HbA1c, TG, TC	Improved glycaemic control
Abbasi et al., 2018 [43]	Double-blind RCT	44 T2DM-DN patients from Iran (case 22; control 22)	Single strain	Probiotic soy milk containing *Lactobacillus plantarum* A7 (2 × 10^7^ CFU/mL)	200 mL/day	8 weeks	↓albuminuria, IL-18, SSA	Improved glomerular function

Discussion

The present study's discussion is based on the results of a thorough analysis of 28 academic publications that looked into how probiotic administration dosages affected important variables in people with T2DM, such as GFR, blood lipid profile, and fasting plasma glucose levels. Of these, 14 articles produced meaningful findings that shed light on the possible therapeutic benefits of probiotics in this particular population.

Promising outcomes were found in two double-blind RCTs among T2DM-DN patients conducted in Iran. The RCTs involved the administration of Lactobacillus plantarum A7 via soy milk. Following an eight-week probiotic intervention, these trials showed significant decreases in sCr levels and increases in eGFR, indicating improved glomerular function [27]. Furthermore, the study's later phases revealed drops in serum sialic acid (SSA), albuminuria, and IL-8 levels, which provides more evidence for *Lactobacillus plantarum* A7's advantageous effects on glomerular filtration [25].

Additionally, after eight weeks of intervention, a parallel RCT examining the effects of *Lactobacillus plantarum* A7 administered through soy milk demonstrated significant reductions in serum levels of cystatin C (Cys-c) and neutrophil gelatinase-associated lipocalin (NGAL). These results point to a possible role for probiotics in modulating tumor necrosis factor-binding protein-1 to reduce inflammation and delay the development of DN [27].

Probiotic treatments using multi-strain microbial cell preparations also showed improvements in glycemic control parameters and renal function markers. One study found that giving patients with T2DM a microbial cell preparation containing different strains of *Lactobacillus acidophilus*, *Lactobacillus casei*, and *Bifidobacterium* spp. significantly reduced their serum urea levels, a sign of improved renal function [31]. Comparably, a daily capsular form of multi-strain microbial cell preparation used in another RCT revealed decreases in fasting plasma glucose, glycosylated hemoglobin, and urinary Alb/Cr, indicating positive effects on renal and glycemic parameters [32].

Further evidence of substantial improvements in a variety of metabolic and inflammatory markers after probiotic supplementation came from a placebo-controlled RCT. Improvements in insulin sensitivity, HDL-c, and plasma GSH levels were noted, along with notable decreases in fasting plasma glucose, serum insulin concentration, HOMA-IR, hs-CRP levels, and MDA levels [29].

Probiotics have been shown to have positive effects on glucose metabolism, lipid profiles, and renal function. This is because they can alter the gut microbiota, which lowers inflammation and oxidative stress. Probiotics affect the host by controlling intestinal permeability, modifying immune responses at the mucosal level, and treating conditions associated with inflammation [44].

Probiotic, prebiotic, and synbiotic formulations in a well-crafted nutraceutical formulation with the right prescription protocols, intervention tactics, targeted population selection, and duration of administration show promise in controlling reno-metabolic markers linked to DN. sCr, BUN, progranulin, soluble tumor necrosis factor receptor 1 (sTNFR1), NGAL, Alb/Cr ratio, IL-18, SSA, and Cys-c are some of these markers. Beneficial effects on renal and metabolic health can result from the synergistic action of probiotics, prebiotics, and synbiotics, which can positively modulate the composition and function of the gut microbiome. Prebiotics specifically promote the growth and activity of good gut bacteria, whereas probiotics aid in the restoration of the gut's microbial balance. Synbiotics are a combination of probiotics and prebiotics that have complementary effects on the gut environment. They improve the survival and activity of probiotic strains. Nutraceutical formulations can help regulate systemic inflammation, oxidative stress, and metabolic dysfunction, all of which contribute to the progression of DN, by influencing gut microbial metabolism and immune function. Particularly, these formulations have the potential to mitigate renal injury by lowering inflammatory markers like IL-18 and sTNFR1, as well as by modifying renal function biomarkers like sCr, eGFR, BUN, Alb/Cr ratio, NGAL, and Cys-c.

Nutraceuticals have a greater influence on metabolic health than just traditional markers of renal function, as evidenced by their possible involvement in controlling novel biomarkers like PGRN, which has been linked to inflammation and insulin resistance. Controlling reno-metabolic markers linked to DN may be possible through the incorporation of probiotic, prebiotic, and synbiotic ingredients into a carefully designed nutraceutical product [45]. Numerous single and multi-strain species combinations of the *Lactobacillus*, *Bifidobacterium*, and *Streptococcus* genera ranging from 5 × 10^6^ to 6 × 10^10^ CFU/day are used for probiotic formulations. Most of the probiotic administration was done through capsules, and very few studies showed the use of mediums like soy milk, yogurt, honey, sachets, and syrup. The multispecies probiotic combination of *Lactobacillus acidophilus ZT-L1*,* Bifidobacterium bifidum ZT-B1*,* Lactobacillus reuteri ZT-Lre*,and* Lactobacillus fermentum ZT-L3 *in capsules led to substantial alterations in BUN, sCr, and eGFR among patients with DN [29]. Three studies covered in a multi-arm, multi-stage trial have shown the use of 200 grams of probiotic soy milk daily for eight weeks containing a single strain of 2 × 10^7^ CFU/mL *Lactobacillus plantarum A7*, which had a prominent and positive impact on the reno-metabolic markers without any adverse effects on the T2DM-DN study group with 44-48 participants [27]. In most of these trials where probiotic administration was used to reduce the progression of DN, the study population was limited; also, very few trials could describe the mechanism of action of the probiotics used on renal function. Therefore, these probiotic formulations cannot be the sole factor responsible for positive renal function. Soy milk naturally has phytochemical properties that, when combined with *Lactobacillus plantarum A7*, favor flavonoids' biological availability by reducing oxidative damage, IL-1, and tumor necrosis factor, improving glomerular functions [45]. Not many studies have shown sustained survival of probiotics in the colon of dysbiotic CKD patients, which is one of the main drawbacks of probiotic therapy [7]. Recent findings indicate that *Lactobacillus plantarum A7 *has a higher chance of surviving in the gut and regulating it, as it can prevail in both acidic and bile environments [45].

These formulations influence the gut microbiome, which causes the production of glucagon-like peptide-1 (GLP-1) and insulinotropic polypeptides. These peptides from the gut are essential for controlling insulin secretion and glucose homeostasis, leading to better glycemic control. While GLP-1 increases insulin secretion and encourages the proliferation of pancreatic beta cells, insulinotropic polypeptides stimulate insulin secretion by pancreatic beta cells. GLP-1 has also been shown to delay stomach emptying, increase satiety, inhibit glucagon secretion, and enhance the absorption of glucose by muscles, all of which improve glycemic control. The synthesis of SCFAs by gut bacteria facilitates this process by providing intestinal epithelial cells with energy and by encouraging the expression of glucose transporters, which in turn facilitates skeletal muscle cells' absorption of glucose [42]. SCFAs that lower the formation of CRP of hepatic origin are formed by probiotics. It reduces cholesterol production, absorption, and plasma cholesterol transport to the liver [46]. These SCFA also have hormone-modulating capacity, especially leptin and ghrelin, which control appetite and blood glycemic levels and maintain body weight. When added to the cumin seed extract, the probiotic soy milk mixture also displays anti-inflammatory properties and regulates blood glucose and lipid levels [47].

## Conclusions

DKD, a severe diabetes mellitus consequence, is known to be influenced by gut microbiota dysbiosis, which contributes to various processes involved in its progression, such as insulin resistance, renin-angiotensin-aldosterone system activation, oxidative stress, inflammation, and stimulating multiple immune responses. The dosage, duration, and combination of probiotic strains chosen for probiotic administration in conjunction with prebiotics and synbiotics in DN, can make this probable adjunct therapy a first-line medical nutritional therapy against DN. Multispecies as well as single-species probiotic administration containing *Lactobacillus*,* Bifidobacterium*,* and Streptococcus thermophilus *spp. greater than 4*10^9^ CFU/day for 8-12 weeks in DN patients improves renal metabolic markers and reduces the progression of disease patterns. More trustworthy results about the effect of probiotic supplementation on DN will come from future clinical trials involving larger populations and time frames.
